# Release of outer membrane vesicles by Gram-negative bacteria is a novel envelope stress response

**DOI:** 10.1111/j.1365-2958.2006.05522.x

**Published:** 2007-01-01

**Authors:** Amanda J McBroom, Meta J Kuehn

**Affiliations:** Duke University Medical Center, Department of Biochemistry Box 3711 Durham, NC 27710, USA.

## Abstract

Conditions that impair protein folding in the Gram-negative bacterial envelope cause stress. The destabilizing effects of stress in this compartment are recognized and countered by a number of signal transduction mechanisms. Data presented here reveal another facet of the complex bacterial stress response, release of outer membrane vesicles. Native vesicles are composed of outer membrane and periplasmic material, and they are released from the bacterial surface without loss of membrane integrity. Here we demonstrate that the quantity of vesicle release correlates directly with the level of protein accumulation in the cell envelope. Accumulation of material occurs under stress, and is exacerbated upon impairment of the normal housekeeping and stress-responsive mechanisms of the cell. Mutations that cause increased vesiculation enhance bacterial survival upon challenge with stressing agents or accumulation of toxic misfolded proteins. Preferential packaging of a misfolded protein mimic into vesicles for removal indicates that the vesiculation process can act to selectively eliminate unwanted material. Our results demonstrate that production of bacterial outer membrane vesicles is a fully independent, general envelope stress response. In addition to identifying a novel mechanism for alleviating stress, this work provides physiological relevance for vesicle production as a protective mechanism.

## Introduction

The capacity of bacteria to mount a multifaceted response to the wide variety of stressors encountered by these organisms *in vivo* and in environmental reservoirs is only recently becoming fully appreciated. Variations in factors such as temperature, nutrient availability, and exposure to toxic agents occur rapidly, requiring an adaptive response for the bacterial cell to survive. In *Escherichia coli*, exposure to damaging stressors can result in the activation of one or more stress responses that are compartmentalized and managed by specialized systems.

Multiple stress response systems monitor and respond to the condition of the envelope compartment. The phage-shock-protein (Psp) system appears to respond to conditions that cause dissipation of the proton motive force, though the method of activation and function of this pathway are not yet clear (Darwin, 2005). The Bae pathway plays a role in resistance to antimicrobial compounds via regulation of multidrug transporters, and shares a number of activating signals with the Cpx response (Raffa and Raivio, 2002). Cpx monitors pili biogenesis by sensing misfolded pilin subunits, and is also involved in surface attachment (Duguay and Silhavy, 2004). In contrast, the σ^E^ pathway is activated in response to misfolded outer membrane proteins (OMPs) (Walsh *et al*., 2003; Wilken *et al*., 2004).

σ^E^ and Cpx are the most well-studied of these stress responses (reviewed in Ades, 2004; Alba and Gross, 2004; Duguay and Silhavy, 2004; Raivio, 2005; Dorel *et al*., 2006). The Cpx pathway functions as a two-component signal transduction system in which an inner membrane-spanning sensor kinase, CpxA, responds to inducing cues by phosphorylating its cognate response regulator, CpxR. Phosphorylated CpxR drives transcriptional regulation of target genes. The σ^E^ pathway is activated upon binding of a periplasmic sensor protease, DegS, to exposed sequences of misfolded OMPs. Signal transduction through this pathway occurs via sequential cleavage of the anti-sigma factor RseA by DegS and RseP, leading to release of the σ^E^ transcription factor and transcriptional activation of a large regulon that includes cell envelope folding and biogenesis factors (Rhodius *et al*., 2005). Activity of the σ^E^ pathway is essential under both stress and non-stress conditions. Also essential at high temperatures is the DegP dual-function protease/chaperone, which is under the transcriptional control of both Cpx and σ^E^ (Danese *et al*., 1995; De Las Penas *et al*., 1997a; Spiess *et al*., 1999). Both Cpx and σ^E^ regulate the expression of folding and degradative factors that act to combat protein misfolding in the periplasm.

In this work we describe a complementary mechanism for managing stress at the cell envelope, production of outer membrane vesicles. Vesicle release by both pathogenic and non-pathogenic Gram-negative bacteria is a ubiquitous process that occurs over the course of normal growth (reviewed in Mayrand and Grenier, 1989; Beveridge, 1999; McBroom and Kuehn, 2005). Native vesicles range from 20 to 300 nm in diameter and are composed exclusively of outer membrane and periplasmic components. Studies of vesiculation by electron microscopy reveal bulging of the outer membrane and subsequent fission of vesicles containing electron-dense material. These biochemical and microscopic observations indicate that vesicles are formed by fission of outer membrane protrusions that enclose periplasmic components.

Once released, vesicles can act as intercellular transport vehicles. Vesicle-associated components may aid in nutrient digestion and elimination of competing organisms (reviewed in McBroom and Kuehn, 2005). Vesicle-mediated delivery of toxins to eukaryotic cells also suggests a role for vesicles in pathogenesis (reviewed in Kuehn and Kesty, 2005). While the composition of vesicles and their interactions with prokaryotic and eukaryotic cells have been investigated, the fundamental physiological role of vesicle production by bacterial cells has remained largely unknown.

The current work demonstrates a novel function for outer membrane vesicle release as a regulated stress response. The process of vesiculation is fully independent from the known stress-responsive systems, and is poised to complement them by offering an effective mechanism for removal of undesirable soluble and insoluble envelope components. Vesicle production is modulated in response to the state of the envelope, decreasing under low stress conditions and increasing with accumulation of overexpressed or misfolded envelope components. Increased vesiculation correlates with increased bacterial survival upon exposure to chemical stressors or accumulation of toxic protein species, and specific packaging of a stress protein mimic into vesicles for removal from the cell demonstrates preferential cargo selection.

## Results

### Characterization of σ^E^ pathway activation levels in vesiculation mutants

**Fig. 1:**
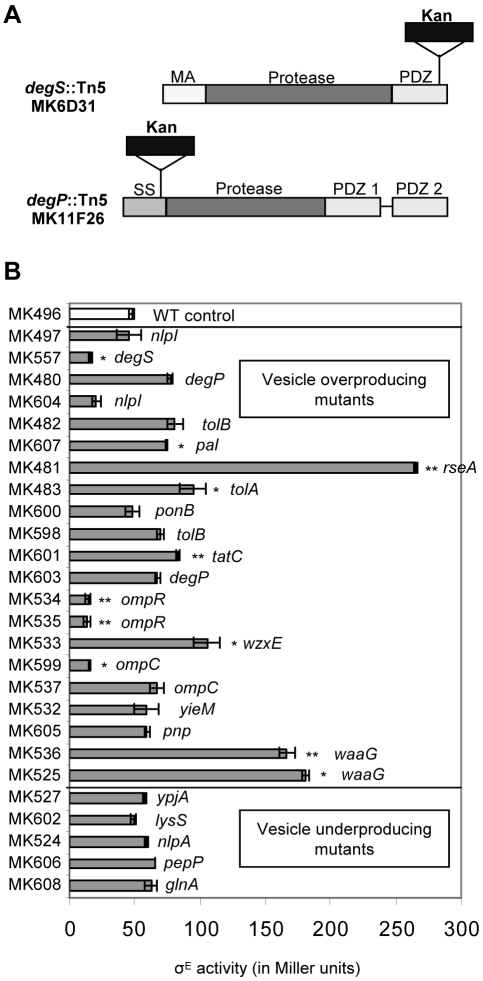
Vesicle production is not directly correlated with activity of the σ^E^ pathway A. Diagrams indicating locations of transposon insertion sites (Kan), membrane anchor (MA), protease, PDZ protein interaction, and signal sequence (SS) domains for the MK6D31 and MK11F26 overvesiculation mutants. The insertion site in MK6D31 would cause an eight-amino-acid truncation of DegS, while the MK11F26 mutant insertion within the signal sequence of *degP* effectively creates a full null. B. β-Galactosidase assays measuring transcription of *lacZ* from the σ^E^-responsive *rpoHP3::lacZ* promoter in ADA600 transduced with mutations affecting vesiculation. ADA600 with pWSK130 (low-copy Kan^R^) is the control. Mutants are listed from the highest vesiculation phenotype (MK8A44 *nlpI*::Tn5) to the lowest (MK9G12 *glnA*::Tn5) based on vesicle OMP quantification (McBroom *et al*., 2006). Cultures were assayed after growth at 37°C to an OD_600_ of 0.3. *P < 0.05, **P < 0.01.

A transposon mutagenesis screen in *E. coli* for factors involved in vesiculation identified overvesiculating mutants with disruptions in the *degS* and *degP* genes of the σ^E^ envelope stress pathway (Fig. 1A). These strains with stress response defects produce approximately 100-fold more vesicles than wild-type *E. coli* without having significant defects in membrane integrity (McBroom *et al*., 2006). This dramatic vesiculation increase in response to impairment of a key stress response pathway provided the first evidence that increased release of vesicles might be compensating for defective stress management.

We initially considered the possibility that vesiculation might be controlled directly by the σ^E^ response. If vesiculation was directly controlled by σ^E^, downregulation of the pathway would decrease vesiculation levels, and activation of the pathway would be required for increased vesiculation. Mutant σ^E^ activity levels were assayed by introducing the vesiculation-altering mutations identified in our screen into a reporter strain expressing *lacZ* from the σ^E^-responsive *rpoHP3* promoter (Fig. 1B). In contrast to this hypothesis, we found that although both mutants overvesiculate, σ^E^ activity was lower than wild-type for *degS*::Tn5, and only very slightly increased in *degP*::Tn5. These results were consistent with previous reports regarding σ^E^ activity in *degS* and *degP* mutants (Mecsas *et al*., 1993; Alba *et al*., 2001).

We continued to examine the relationship between σ^E^ and vesiculation by examining σ^E^ levels in all of our previously characterized vesiculation mutants. While we have predicted previously that many of these strains have altered vesiculation levels for reasons entirely unrelated to stress (McBroom *et al*., 2006), we wished to determine whether any correlations between σ^E^ activity and vesiculation could be drawn. Strains with disruptions in *waaG*, *wzxE*, *tatC*, *pal* and *tolA* exhibited statistically significant σ^E^ activity increases, while σ^E^ activity in *ompR* and *ompC* mutants was decreased. These results agree with reports of altered σ^E^ pathway behaviour in strains that have decreased expression of the OmpF and C porins (*ompR* and *ompC*), abnormal LPS (*waaG*), or polar disruptions of *wxzE* (Mecsas *et al*., 1993; Missiakas *et al*., 1996; Danese *et al*., 1998). We also observed that the σ^E^ pathway is constitutively active in *rseA*::Tn5, consistent with previously published reports regarding σ^E^ activity in *rseA* mutants (De Las Penas *et al*., 1997b; Missiakas *et al*., 1997). Though these mutations all cause vesicle overproduction, their effects on σ^E^ activity levels varied. Further, the σ^E^ activity levels of the vesicle underproducing strains did not deviate significantly from wild type (Fig. 1B). Thus, a comparison between mutant vesicle production phenotypes and σ^E^ activation states demonstrates that there is no direct correlation between σ^E^ activity level and quantity of vesicle release. Vesiculation levels must be regulated by other means.

### Vesiculation increases in response to impairment of the σ^E^ pathway

**Fig. 2:**
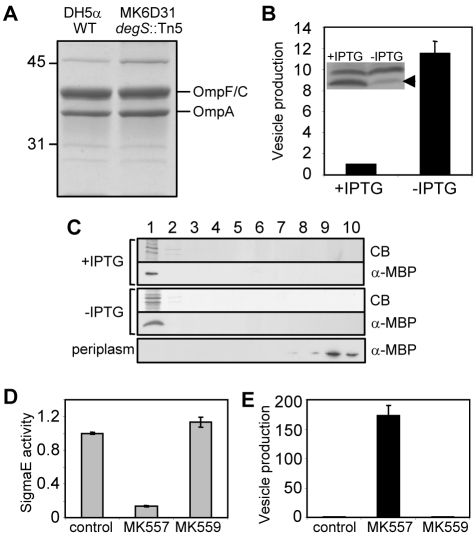
Reduced DegS function impairs σ^E^ activation and increases vesicle production A. Porin profiles of purified outer membranes prepared from OD-matched cultures of wild-type DH5α and MK6D31 *degS*::Tn5. Samples from equal culture densities were assayed by SDS-PAGE and Coomassie Blue staining. B. Vesicle production of DegS-depletable strain CAG43248 grown with (DegS expressed) or without (DegS depleted) IPTG. Values are relative to the culture grown with IPTG. (Inset) DegS depletion in the absence of IPTG shown by immunoblot of equivalent amounts of cell lysates at vesicle harvest. DegS is indicated (arrowhead); the upper band is a non-specific loading control. C. Equilibrium density gradients of vesicles from DegS-expressed and DegS-depleted cultures. Periplasm from DH5α was applied to the same gradient as a control for non-vesicle-associated MBP. Fraction 1 is the least dense; fraction 10 is the most dense. Prominent vesicle proteins were visualized by Coomassie Blue (CB) stained SDS-PAGE; MBP was visualized by immunoblotting (α-MBP). D and E. σ^E^ activity levels at the time of vesicle harvest (D) and vesicle production (E) of wild-type ADA600 and *degS*::Tn5 transductants MK557 and MK559. Values are relative to the ADA600 control.

The *degS*::Tn5 vesicle overproducing mutant displayed significant σ^E^ activity reduction (Fig. 1B). We needed to be cautious in interpreting this phenotype because a number of genes implicated in the insertion of β-barrel proteins into the outer membrane have σ^E^ promoters (Rhodius *et al*., 2005). In addition, *degS* is essential, and strains lacking it develop suppressor mutations at a high rate (Alba *et al*., 2001). We first considered the possibility that the overvesiculation of *degS* mutants with reduced σ^E^ activity could be caused by an outer membrane porin deficiency. SDS-PAGE analysis of purified outer membrane fractions demonstrated that this is not the case, as the outer membrane of the *degS* mutant does not have a reduced porin content (Fig. 2A).

Further, we noted that the location of the Tn5 insertion in our *degS* mutant would cause only an eight-amino-acid truncation (Fig. 1A). While slight, this truncation lies in the stress-sensing DegS PDZ domain. To avoid potential complications from suppressing mutations in our analysis, we used a DegS-depletable strain (CAG43248) to test the vesiculation effect of a controlled reduction in DegS activity. This strain carries an isopropyl-β-D-thiogalactopyranoside (IPTG)-inducible promoter preceding the chromosomal *degS* gene and requires IPTG for *degS* expression. After a 4 h period of growth without IPTG, viability was not lost (Fig. S1), and depletion of DegS was confirmed (Fig. 2B, inset). Vesicle production assays after 4 h of growth in the presence or absence of IPTG demonstrated increased vesiculation by the DegS-depleted culture (Fig. 2B).

Due to the relationship between σ^E^ promoters and outer membrane composition (Rhodius *et al*., 2005), we also wished to verify that the vesicles produced under these conditions were released as intact structures. Vesicles produced under both conditions were subjected to equilibrium density centrifugation. Vesicles migrate to light density fractions, while non-vesicle-associated material remains in the heavy fractions. To test for vesicle integrity, fractions from the gradients were immunoblotted to detect periplasmic maltose binding protein (MBP). MBP, which would only be present in the interior of intact vesicles, co-migrated with OMPs to the light density fractions for vesicles produced under both culture conditions (Fig. 2C). Non-vesicle-associated MBP remained in the bottom fractions. These results indicate that the vesicles produced under DegS-depletion contain lumenal periplasmic material, thus confirming their intact nature.

Transduction of the *degS*::Tn5 mutation obtained in our screen into ADA600 yielded MK557, a transductant with reduced σ^E^ activity (Figs 1B and 2D). However, we also isolated a transductant with σ^E^ activity near wild type, which we named MK559 (Fig. 2D). Sequencing confirmed incorporation of the transposon disruption at the correct site in both strains. Both transductants may carry secondary mutations that suppress the essentiality of DegS; however, MK559 must carry a unique mutation that restores its σ^E^ activity to wild-type levels. To determine if the difference in σ^E^ activity between these two transductants would correspond to a difference in vesiculation phenotype, we assayed the two strains for levels of vesicle release. Similar to our original *degS*::Tn5 mutant (MK6D31), the transductant with reduced σ^E^ activity overproduced vesicles to a high degree; in contrast, the transductant with wild-type σ^E^ activity released vesicles at wild-type levels (Fig. 2E). Together, the results demonstrate that the increased vesiculation phenotype observed when DegS is depleted or mutated correlates with decreased σ^E^ responsiveness. Such impairment in DegS function would result in the accumulation of improperly folded species in the periplasm (Mogensen and Otzen, 2005).

### Vesiculation increases with temperature-induced stress

**Fig. 3:**
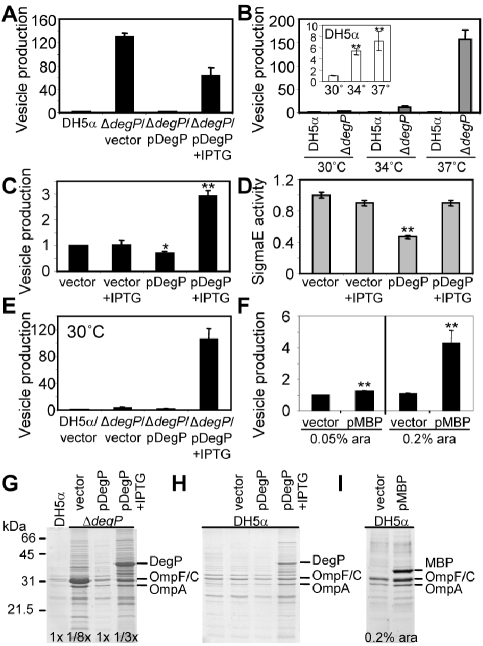
Vesicle production is modulated in response to envelope stress A. Vesicle production of DH5α and MK11F26 *degP*::Tn5 with no plasmid, vector (pCS19), or DegP plasmid (pCS20) grown at 37°C. Where indicated, cultures contained 10 μM IPTG; values are relative to DH5α. B. Vesicle production of DH5α and MK11F26 *degP*::Tn5 at 30°C, 34°C and 37°C. Each set is normalized to the DH5α control for that temperature. (Inset) Vesicle production of DH5α at 30°C, 34°C and 37°C; values are relative to the 30°C condition. C. Vesicle production of DH5α with vector (pCS19) or DegP plasmid (pCS20) at 37°C. Where indicated, cultures contained 10 μM IPTG; values are relative to DH5α with vector. D. σ^E^ activity at the time of vesicle harvest in cultures of ADA600 corresponding to conditions in C. Values are relative to ADA600 with vector. E. Vesicle production of MK11F26 *degP*::Tn5 with vector (pCS19) or DegP plasmid (pCS20) at 30°C. Where indicated, cultures contained 10 μM IPTG; values are relative to DH5α with vector. F. Vesicle production of DH5α with vector (pHDB67) or MBP expression plasmid (pJH68). Cultures contained 0.05% or 0.2% arabinose; values are relative to DH5α with vector at the corresponding induction level. For all panels *P < 0.05, **P < 0.01 are indicated for instances where differences are not obviously significant. G–I. Samples of vesicles from cultures described in A, C and F (0.2% arabinose induction), respectively, analysed by SDS-PAGE and Coomassie Blue staining. Bands corresponding to OMPs F/C and A, DegP and MBP are labelled; loading volume dilution factors in G are indicated.

Another overvesiculating mutant that we could identify as directly impaired in ability to cope with accumulated envelope stress products was *degP*::Tn5. Mutants lacking DegP are impaired in chaperone and protease activity and accumulate misfolded envelope proteins with rising temperatures (Clausen *et al*., 2002). The MK11F26 *degP*::Tn5 mutant exhibits a strong overvesiculation phenotype, and we verified that complementation with a *degP* plasmid (pCS20) reduced the strain's vesiculation phenotype > 100-fold to near wild-type levels (Fig. 3A). We hypothesized that if the accumulation of misfolded proteins triggers vesicle production in the *degP* mutant, the vesiculation phenotype of the strain should vary directly with temperature. In Fig. 3B, vesiculation levels for *degP*::Tn5 are normalized to the isogenic parent for each of three temperature conditions in order to demonstrate the difference between mutant and wild-type behaviours within each temperature set. While *degP*::Tn5 exhibits a very high level of vesicle production relative to wild type during growth at 37°C, its phenotype is similar to wild type when both are grown at 30°C. Growth at an intermediate temperature of 34°C results in an intermediate vesiculation phenotype. Thus, growth temperature dramatically impacts the difference in relative quantity of vesicle production by *degP*::Tn5.

As temperature-induced protein misfolding also occurs in wild-type cells, we tested whether thermal induction of vesiculation was limited to the mutant strain. In fact, it was not. Wild-type DH5α also responded to increasing temperature by releasing a larger quantity of vesicles (Fig. 3B, inset). This increase is far less dramatic than that observed for the *degP* mutant because DegP activity in the wild-type strain reduces the protein misfolding effects of thermal stress. The influence of temperature upon vesicle production by the *degP*::Tn5 and wild-type strains suggests that enhanced vesiculation at higher temperatures is a response to misfolded protein accumulation.

### Vesiculation is regulated by the level of protein accumulation in the envelope

Whereas complementation with uninduced DegP restored *degP*::Tn5 vesiculation levels to wild type, we observed a heightened vesiculation response to induced overexpression of DegP in this strain (Fig. 3A, compare Δ*degP*/pDegP and Δ*degP*/pDegP + IPTG). We also noted that the vesiculation increase corresponding to induced DegP overexpression in the wild-type strain was less pronounced than in the mutant (Fig. 3C, pDegP + IPTG). To determine whether the difference in vesiculation levels between the two strains correlated with a difference in DegP production, we immunoblotted OD-matched total culture samples to compare the quantity of overexpressed material. In the *degP* mutant background, IPTG induction of pCS20 caused a 40-fold increase in total DegP quantity over that of DH5α and the vector controls, while induction in the DH5α background gave an approximately 10-fold increase (data not shown). Correspondingly, DegP overexpression in the mutant caused a 60-fold increase in vesicle production, while the increase in the wild-type strain was approximately threefold (Fig. 3A and C; compare + IPTG values). Thus, the extent of vesiculation correlated with the magnitude of protein overexpression.

These results suggest that overexpression of DegP should also be able to drive heightened vesiculation in *degP*::Tn5 grown at 30°C, a temperature at which the mutant produces a quantity of vesicles similar to the wild-type strain. Indeed, induced overexpression of DegP caused a dramatic increase in vesicle production under these conditions (Fig. 3E; Δ*degP*/vector, Δ*degP*/pDegP, Δ*degP*/pDegP + IPTG).

We further hypothesized that the vesiculation response to periplasmic protein overexpression should be independent of the identity of the overexpressed polypeptide. To test this, we assayed the vesiculation of DH5α upon induced overexpression of plasmid-encoded periplasmic MBP. Expression of MBP from this plasmid is under the control of an arabinose-inducible promoter. Vesiculation increased by 1.3-fold relative to the induced vector control at an arabinose concentration of 0.05%; at a higher induction level of 0.2% arabinose, vesicle production increased by fourfold (Fig. 3F). These results demonstrate the dose-dependent general nature of the vesiculation response to bulk periplasmic protein overexpression.

Analysis of vesiculation levels in response to a modest increase in *degP* expression revealed an opposite, subtle modulation effect. Uninduced DegP expression from the pDegP plasmid in wild-type DH5α increased the total quantity of expressed DegP by 1.4-fold (data not shown). Correspondingly, vesicle production decreased by 30% (Fig. 3C; compare vector, pDegP). Vesicle release was unaffected in controls for the presence of vector and IPTG. We moved the vector and inducible DegP plasmid into the σ^E^ reporter strain to examine the effect of low-level DegP expression on envelope stress. While the σ^E^ pathway does not monitor all types of stress (reviewed in Ruiz and Silhavy, 2005), its activation level does provide a useful assessment of the state of the envelope. We observed that σ^E^ activity in cells expressing low levels of exogenous DegP was significantly lower than wild type (Fig. 3D; compare vector, pDegP). Therefore, the vesiculation decrease caused by slightly elevated DegP levels correlates with a reduction in envelope stress, presumably due to the slight increase in DegP chaperone/degradative activity in the envelope. This result demonstrates another important aspect of regulation. Not only does vesiculation increase upon heightened stress, it can also be downregulated upon a reduction in the stress state of the cell envelope.

### Increased vesiculation improves bacterial survival under stress

**Fig. 4:**
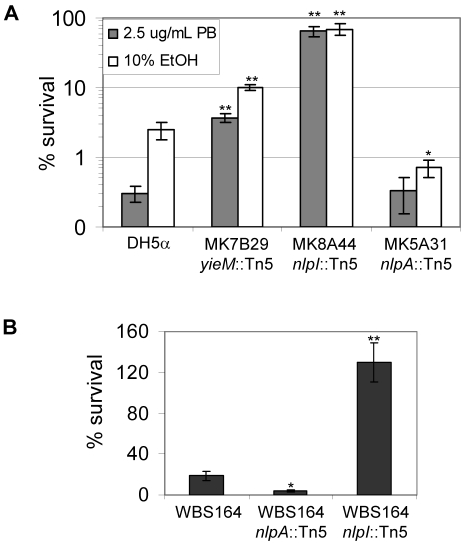
Increased vesicle production enhances bacterial survival under stress A. Survival of wild-type DH5α, a mutant with moderately increased vesiculation (MK7B29 *yieM*::Tn5), a mutant with highly increased vesiculation (MK8A44 *nlpI*::Tn5), and a mutant with reduced vesiculation (MK5A31 *nlpA*::Tn5) after 2 h of exposure to 10% ethanol (white bars) or 2.5 μg ml^−1^ polymyxin B (grey bars). B. Survival of WBS164 carrying the maltose-inducible *lamB-lacZX90* allele in comparison with transductants of WBS164 with either the *nlpA*::Tn5 mutation causing vesicle underproduction or the *nlpI*::Tn5 mutation causing vesicle overproduction. Expression of the toxic LamB-LacZX90 fusion protein was induced for 2 h with 0.02% maltose. For both panels *P < 0.05, **P < 0.005.

The data presented thus far are consistent with a model in which vesicle production is a mechanism for the bacterial cell to combat misfolded and accumulated envelope protein stress. Based on this model, we hypothesized that the mutant strains found in our previous genetic screen which over- or under-produce vesicles would exhibit an altered response to challenge with envelope-stressing agents. We used a stringent selection criteria for these experiments, choosing mutants with no observed defects in membrane integrity, no known link to previously established stress pathways, no obvious changes to the outer membrane, and no significant difference from wild type in σ^E^ activity assays. MK7B29 *yieM*::Tn5, which produces approximately 7.5-fold more vesicles than DH5α, and MK8A44 *nlpI*::Tn5, which produces over 100-fold more, fit these criteria (Nishino *et al*., 2005; Rhodius *et al*., 2005; Dorel *et al*., 2006; McBroom *et al*., 2006) (Fig. 1B). We also confirmed that the MK8A44 *nlpI*::Tn5 disruption does not increase activity of the Cpx or Bae stress pathways (data not shown). We calculated the per cent survival of wild-type and mutant strains after a 2 h period of exposure to 10% ethanol or the outer membrane-damaging antimicrobial peptide polymyxin B. The overvesiculating mutants exhibited enhanced survival in a pattern that mimics their vesiculation phenotypes; the increase in stressor resistance was moderate for MK7B29, and markedly higher for the highly overvesiculating MK8A44 (Fig. 4A). In contrast, MK5A31 *nlpA*::Tn5, a vesicle underproducing mutant with no membrane integrity defects (McBroom *et al*., 2006), performed poorly in both survival assays (Fig. 4A). In addition to these selected strains, we tested the entire panel of vesicle overproducing mutants for survival upon polymyxin treatment. With a few exceptions, such as the *tatC* and *ponB* mutants with extensive pleiotropic defects and *degP* mutants with stress response defects (McBroom *et al*., 2006), we observed a general trend of increased survival with increased vesiculation levels (data not shown).

While the polymyxin and ethanol assays demonstrate that increased vesiculation correlates with enhanced resistance to lethal envelope stress, we wished to specifically test the ability of vesiculation to alleviate damage to the cell due to accumulation of misfolded envelope proteins. Therefore, we tested the ability of vesiculation to relieve toxicity caused by expression of the LamB-LacZX90 fusion protein, a misfolded species whose accumulation in the periplasm is lethal (Snyder and Silhavy, 1995). We introduced mutations causing either increased or decreased vesicle production into WBS164, the parental strain carrying the maltose-inducible chromosomal *lamB-lacZX90* allele. Mid-log phase cultures were washed to remove any vesicles produced to that point and resuspended in fresh media with maltose to induce LamB-LacZX90 expression. As shown in Fig. 4B, the strain with decreased vesiculation had a significant decrease in survival, while the survival of a mutant with increased vesiculation was dramatically improved. This finding demonstrates that vesicle production is a mechanism for the cell to protect itself from the toxic accumulation of misfolded proteins.

### Vesiculation is a distinct, independent stress response

Our data reveal that the production of outer membrane vesicles is upregulated upon impairment of σ^E^ pathway function but is not directly controlled by σ^E^ levels. The effects of stress in the bacterial cell envelope are monitored not only by σ^E^, but also by the Psp, Cpx and Bae systems. To determine whether vesiculation is a fully independent stress response mechanism, we assessed the potential of the other known extracytoplasmic stress responses to regulate vesicle production. Considering that inactivation of these pathways might affect the marker proteins we typically use for vesicle quantification (OMPs F, C and A), we used both total lane protein densitometry and a lipid reagent-based assessment technique that employs the lipid probe FM4-64 (McBroom *et al*., 2006) in addition to OMP-based quantification methods. These three methods of quantification all gave results that were in close agreement; therefore, we have presented only the OMP data.

**Fig. 5:**
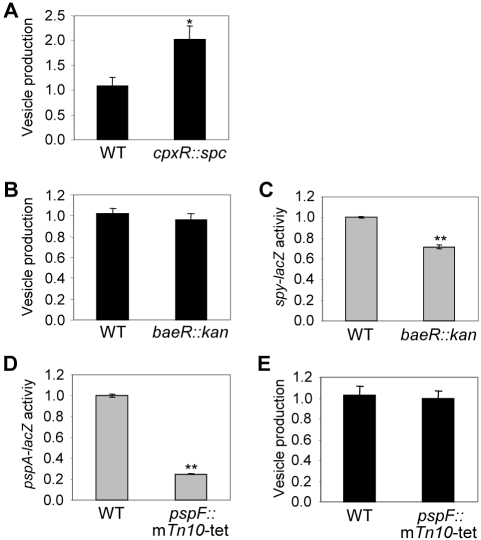
Vesiculation is fully independent of all other known envelope stress responses A. Vesicle production of TR51 (*cpxR*::*spc*) relative to its isogenic parent MC4100 (WT). B and C. Vesicle production (B) and *spy-lacZ* promoter activity at the time of vesicle harvest (C) of TR886 (*baeR*::*kan*) relative to its isogenic parent TR530 (WT). *spy* promoter activity is only partially regulated by the Bae pathway, but the reduction in activity is apparent. D and E. Psp pathway activity as measured by *pspA-lacZ* promoter activity at the time of vesicle harvest (D) and vesicle production (E) of MK789 (*pspF*::mTn*10-tet*) relative to its isogenic parent MVA4 (WT). For all panels *P < 0.05, **P < 0.005.

The Cpx and Bae pathways are typical two-component signal transduction systems. Deletion of *cpxR* encoding the response regulator of the Cpx pathway causes a dramatic decrease in transcription of Cpx-regulated genes (Raivio *et al*., 1999). If vesicle production is directly controlled by the Cpx pathway, the *cpxR* null mutant would exhibit a corresponding decrease in vesiculation. In our assays comparing vesicle production by the *cpxR* mutant to its isogenic wild-type parent, we show instead that impairment of the Cpx response results in a vesiculation increase (Fig. 5A). In a similar fashion, we evaluated the vesicle production phenotype of a *baeR* mutant strain. Deletion of the Bae pathway response regulator did not alter vesiculation levels (Fig. 5B). Decreased Bae-regulated transcription in the *baeR* mutant was confirmed by a reduction in activity from a *lacZ* fusion to a promoter that is partially regulated by Bae (Fig. 5C). These data demonstrate that vesiculation is not directly controlled by the Cpx or Bae pathways, and that, in fact, an impaired Cpx pathway may lead to accumulation of envelope stress products similar to impaired stress sensing by the σ^E^ pathway.

Activity of the Psp response can be greatly reduced by a *pspF*::mTn*10-tet* disruption (Jovanovic *et al*., 1996). PspF is the transcriptional activator of the pathway, and this disruption results in expression of a PspF truncate incapable of binding to DNA at physiological concentrations (Jovanovic *et al*., 1996). The *pspF*::mTn*10-tet* mutant and isogenic parent strain used in our studies carry a *lacZ* reporter fusion to the *pspA* promoter, which is under the control of PspF-driven transcription. Assays at the time of vesicle harvest confirmed a significant loss of Psp pathway transcriptional activity in the *pspF*::mTn*10-tet* mutant (Fig. 5D), but did not reveal any differences in vesicle production (Fig. 5E). Therefore, vesiculation is a stress response mechanism that is independent and distinct from all previously known envelope stress responses.

### Packaging of stress products into vesicles

Vesiculation offers bacterial cells the ability to export soluble and insoluble stress products. Inclusion of overexpressed periplasmic proteins as major vesicle components would demonstrate that material causing stress in the envelope can be packaged as cargo into vesicles and exported out of the cell. Indeed, DegP is clearly visible in protein profiles of vesicles produced by strains overexpressing plasmid-encoded DegP (Fig. 3G and H). The identity of this band was established by immunoblotting, and DegP association with intact vesicles was confirmed by co-migration with OMPs in equilibrium density centrifugation fractions (data not shown). The same phenomena can be observed in the protein profiles of vesicles produced by bacteria overexpressing plasmid-encoded MBP (Fig. 3I). The ratio of highly overexpressed DegP to endogenous MBP in vesicles and periplasm did not indicate enrichment of DegP in the overproduced vesicles (data not shown).

**Fig. 6:**
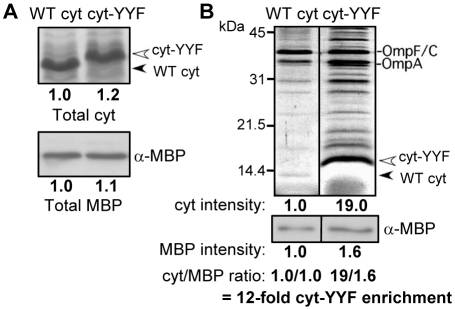
Vesiculation allows preferential packaging and elimination of cargo from the cell envelope A. Representative total OD-matched culture samples of wild-type *rpoHP3::lacZ* reporter strain CAG16037 expressing WT cyt or cyt-YYF assayed by SDS-PAGE and immunoblotting for cytochrome (upper panel) and MBP (lower panel). Relative densitometry values for cytochrome and MBP content are indicated. The quantity of each species present in the WT cyt-expressing culture sample is set to 1.0. B. Representative vesicles from cultures of CAG16037 expressing WT cyt and cyt-YYF analysed by SDS-PAGE and Coomassie Blue staining (upper panel) or immunoblotting for MBP (lower panel). Relative densitometry values and cyt/MBP ratios are indicated. Experimental average for fold cyt-YYF enrichment is 10.2 ± 1.5 SEM.

Though a bulk-flow mechanism of material export from the periplasm may be sufficient to reduce stress in the envelope, it seems that it would be most advantageous for the cell to selectively dispose of damaged or misfolded proteins by loading them into vesicles while retaining functional, properly folded polypeptides. To determine whether damaged or misfolded proteins can be selectively packaged into vesicles, we assessed the ability of the cell to preferentially incorporate a construct designed to mimic a misfolded envelope protein. This polypeptide is composed of periplasmic cytochrome b_562_ fused to 50 amino acids of OmpC and terminating in the sequence YYF (abbreviated cyt-YYF). The cyt-YYF construct mimics a misfolded OMP intermediate that has not been properly inserted into the outer membrane, and is recognized by the cell as a σ^E^-activating stress signal (Walsh *et al*., 2003). We selected MBP, a native periplasmic component, as an endogenous reference cargo control. We compared the ratio of incorporation into vesicles for native MBP, which can be presumed to be properly folded, to either cyt-YYF or wild-type cytochrome b_562_ (WT cyt). The WT cyt construct provides a control for protein overexpression that does not contain an OMP-like stress signal; however, MBP, not WT cyt, was used as a reference cargo to determine selective enrichment because WT cyt is not expressed at native levels. We first verified that similar amounts of cytochrome products were produced by the strains. OD-matched total culture samples of CAG16037 expressing either WT cyt or cyt-YYF were analysed by SDS-PAGE. Relative densitometry values illustrate that the two cytochrome products are produced at similar quantities (Fig. 6A). Immunoblotting of these samples also showed that MBP levels between the two strains did not vary (Fig. 6A).

Vesicles produced by WT cyt or cyt-YYF-expressing cultures were then analysed by protein staining and immunoblotting to compare their internal ratios of cytochrome construct to MBP (Fig. 6B). Vesicle sample volumes containing similar amounts of MBP were subjected to SDS-PAGE and protein staining, and the cytochrome bands quantified by densitometry. While the WT cyt control peptide and cyt-YYF fusion are both packaged into vesicles, the ratio of cytochrome incorporation relative to MBP for cyt-YYF is substantially higher. This ∼10-fold enrichment for cyt-YYF in vesicles suggests a selective mechanism for the inclusion of specific cargo. The overall increase of other proteins in the cyt-YYF vesicles as compared with the WT cyt vesicles may be due to production of envelope stress response factors stimulated by expression of this construct. It is also possible that overexpressing cyt-YYF might titrate periplasmic folding factors or proteases away from their typical substrates. Titration of this type could lead to increased levels of misfolded OMPs and periplasmic proteins, which would be potential candidates for specific packaging into vesicles. In comparison with the strain expressing WT cyt, expression of cyt-YYF resulted in a 10-fold increase in σ^E^ activity as well as an approximately 10-fold increase in vesiculation level. This indicates that increased vesiculation levels and a specific packaging mechanism act in concert to amplify removal of the stress product.

### *Vesiculation is also a response to stress in* Salmonella

**Fig. 7:**
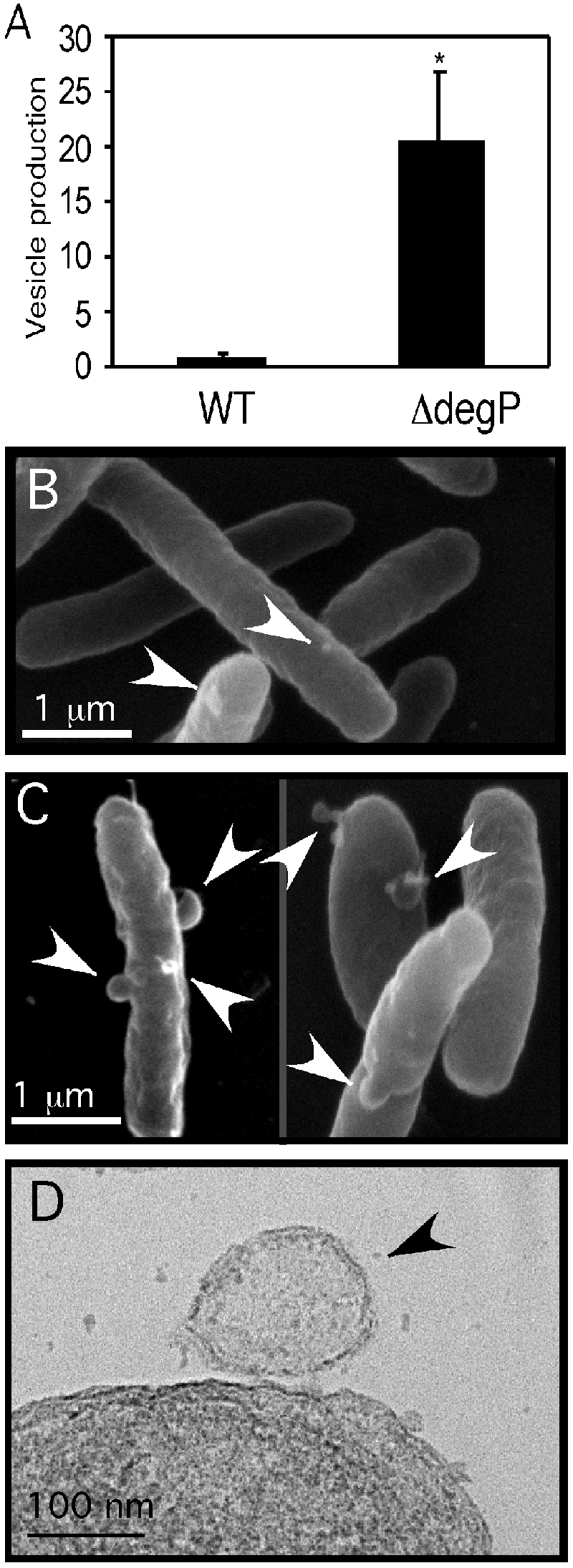
Stress-induced vesiculation in Gram-negative bacteria A. Vesicle production of WT *Salmonella* ATCC 14028 and ATCC 14028 *degP*::cat grown at 37°C; values are relative to WT. *P < 0.05. B and C. Scanning electron micrographs of DH5α (B) and MK11F26 *degP*::Tn5 (C) grown at 37°C. D. Thin-section transmission electron microscopy of *degP*::Tn5 grown at 30°C overexpressing DegP from pCS20 induced with 10 μM IPTG. Vesicles are indicated (arrows).

After exploring the link between envelope stress and vesicle release in *E. coli*, we wished to determine whether this link also existed in other Gram-negative bacteria. To study the effect of misfolded protein accumulation, we compared the vesiculation phenotype of wild-type pathogenic *Salmonella enterica* serovar Typhimurium to an isogenic *degP* mutant derivative thereof (Fig. 7A). As predicted, loss of *degP* increased vesiculation. Thus, our results from studies in *E. coli* extend to the broader Gram-negative bacterial population.

## Discussion

Although production of outer membrane vesicles by Gram-negative bacteria has been described by microscopic observations for decades, only recently have genetic and biochemical studies begun to reveal its physiological utility. Far from an artifact of experimental design or a by-product of imbalanced cell division, vesicle release is a specific mechanism for secretion of envelope components. Production of vesicles offers the cell an effective method for the elimination and remodelling of envelope components, particularly during times of stress. The data presented in this work demonstrate that vesiculation is an independent, flexible process for stress management.

The quantity of material released as vesicles is positively and negatively regulated in direct correlation with the degree of stress product build-up in the envelope. For example, a slight increase in DegP expression increases the housekeeping capacity of the envelope, lowering the quantity of misfolded products and consequently reducing the need for the cell to release this toxic material in vesicles. Conversely, the DegP protein itself becomes an unwelcome envelope component when overexpressed at a high level in the periplasm, and the cell increases vesiculation to expunge the additional material. The same response is observed upon overexpression of increasing quantities of MBP. Thus, the increased vesiculation response is both general and dose-dependent.

The DegS truncate produced by the MK6D31 and MK557 *degS*::Tn5 mutants is presumably unstable or impaired in binding to misfolded OMPs, rendering it sensing deficient. Impaired stress sensing and an inability to upregulate extracytoplasmic stress response factors in these mutants would result in the accumulation of misfolded envelope proteins (Mogensen and Otzen, 2005). Similarly, *degP* mutants are unable to effectively reduce the normally produced levels of misfolded envelope proteins. DegP becomes essential at high temperatures and is the primary protease responsible for degradation of misfolded proteins in the periplasm (Clausen *et al*., 2002). An *lpp*^–^ mutation that causes outer membrane instability and leakage of periplasmic proteins suppresses *degP*^–^ high-temperature lethality, in theory by allowing stress products to leak out of the cell (Strauch *et al*., 1989). The heightened vesiculation phenotype of the *degP* mutant is stress-dependent, increasing with higher temperatures that amplify protein misfolding. We propose that *degP* mutants use increased vesiculation as a survival mechanism to rid the cell of toxic misfolded proteins under sublethal stress conditions. This appears to be a common mechanism, as vesiculation of uropathogenic *E. coli* increases upon deletion of the gene encoding SurA, a periplasmic OMP chaperone (A. McBroom, D. Hunstad and M. Kuehn, unpubl. data). OMP misfolding is elevated in *surA* mutants (Missiakas *et al*., 1996; Rouviere and Gross, 1996).

The vesiculation process is not controlled by any of the previously described envelope stress responses. Instead, impairment of either σ^E^ or Cpx results in increased vesicle production. σ^E^ and Cpx appear to be the primary signal transduction pathways responsible for monitoring and responding to envelope protein misfolding. When these systems are impaired, the cell is unable to properly manage protein misfolding and aggregation in the envelope compartment. Potentially damaging material accumulates, and vesicle production is upregulated to compensate for the inability of the cell to fully address folding imbalances. Vesiculation in response to the bulk accumulation of material in the envelope is not mediated by σ^E^, because the increase in vesiculation upon the overexpression of DegP or MBP occurs without an increase in σ^E^ activity (Fig. 3D and Mecsas *et al*., 1993). Although vesiculation is independent of known envelope stress pathways, recent work in our laboratory indicates that σ^E^ pathway stimulation is sufficient to increase vesiculation (A. J. McBroom and M. J. Kuehn, unpubl. data). At first glance, this would seem to be a paradoxical finding. However, we propose that in such situations, vesiculation increases as a result of increased envelope products: either an increase of σ^E^-regulated proteins accumulating in the envelope, an increase in proteolytic products in the envelope due to σ^E^-induced protease activity, and/or the accumulation of the σ^E^ pathway stimulus in the envelope.

We studied whether the incorporation of lumenal cargo into vesicles is selective or occurs by bulk-flow. Preferential vesicle packaging of the cyt-YYF stress protein analogue relative to MBP (Fig. 6B) suggests that misfolded protein cargo may be selectively enriched in vesicles. However, selective packaging does not appear to occur for all vesiculation-stimulating cargo: an increase in the quantity of the stressor product in the periplasm also leads to a correspondingly high level of incorporation into vesicles, suggestive of bulk-flow movement of protein into these structures (Fig. 3G–I). Either selective or bulk-flow methods of cargo loading coupled with increased vesicle production would enable removal of toxic material from the cell envelope.

Our data testing the relationship of vesiculation to protein misfolding and overexpression, thermal stress and modulation of the folding capacity of the envelope can be distilled to a unifying model in which vesicle production serves to maintain a periplasmic content ‘equilibrium’. In this respect, it is not surprising that maximal vesicle production occurs during exponential growth (Bauman and Kuehn, 2006, reviewed in McBroom and Kuehn, 2005), a period in which cells likely experience the largest disturbance of this equilibrium due to high levels of protein expression, folding and misfolding. A build-up of damaged proteins may lead to distension of the periplasm and subsequent bulging of material into vesicles, or the accumulated misfolded material may upregulate vesicle production through a more indirect means. Cultures of wild-type and vesicle-overproducing strains examined by scanning and transmission electron microscopy revealed intact membranes and vesicles with similar diameters (20–200 nm) (Fig. 7B–D), suggesting a common physical mechanism for vesicle-mediated stress relief.

Release of outer membrane vesicles offers the cell an effective mechanism for removal of material as a macromolecular complex, allowing the cell to discard unwanted material or alter the composition of the envelope under conditions where remodelling would be advantageous. Bacteria, particularly pathogens, encounter a variety of envelope stresses. Vesicle production offers a mechanism for the cell to protect itself from such insults. For example, antimicrobials can bind to vesicle decoys surrounding the cell, or they can bind to the outer membrane and become detached upon vesicle release. Our chemical, antibiotic and lethal protein challenges of vesiculation mutants demonstrate that vesiculation correlates positively with survival.

Transport of material via membrane-bound vesicles is a common phenomenon for eukaryotic cells, and it is clear that this capability extends to prokaryotes as well. An interesting parallel can be drawn between vesiculation by Gram-negative bacteria and the release of vesicles (called ectosomes or microparticles) formed from the plasma membrane of eukaryotic cells. Microparticle release has been observed for a wide range of eukaryotic cell types in response to activating stimuli (Martinez *et al*., 2005). Eukaryotic cells also release exosomes, which are endosomal in origin and whose release appears to increase upon heat stress (Clayton *et al*., 2005). Activities attributed to eukaryotic vesicles include involvement in angiogenesis, development and immune response, as well as specific elimination of undesirable components from the cell (Thery *et al*., 2002; Pilzer *et al*., 2005).

Our work demonstrating regulated, specific release of envelope material via outer membrane vesicles extends this paradigm to Gram-negative bacteria and fulfil the proposed key requirements of a genuine prokaryotic secretion process (Economou *et al*., 2006). Further work is required to identify how envelope stress transmits into the mechanics of outer membrane bulging and fission, as well as the mechanism of cargo selection.

## Experimental procedures

### Growth conditions and reagents

Strains and plasmids are described in Table 1. Bacterial strains were grown in Luria–Bertani (LB) broth (EM Science) or on LB agar supplemented with 50 μg ml^−1^ kanamycin, 100 μg ml^−1^ ampicillin or 25 μg ml^−1^ chloramphenicol (Sigma). Arabinose (Sigma) or IPTG (VWR) was added to induce protein expression. Transductions were performed with P1 phage (Silhavy *et al*., 1984). Antibodies were purchased (MBP, NEB) and kindly provided by J. Beckwith (DegP-MBP) and C. Gross (DegS).

**Table 1:** Strains and plasmids.

Strain/plasmid	Relevant genotype/description	Source/reference
Strains		
DH5α	F^–^φ80*lacZ*ΔM15 Δ(*lacZYA-argF*)U169 *deoR recA endA1 hsdR17*(r_k_^–^, m_k_^+^) *phoA supE44 thi-1 gyrA96 relA1*λ^–^	Gibco
MK6D31	DH5α*degS*::Tn5, insertion after codon 347 of 355	McBroom *et al*. (2006)
MK11F26	DH5α*degP*::Tn5, insertion after codon 22 of 474	McBroom *et al*. (2006)
MK5B7	DH5α*rseA*::Tn5, insertion after codon 168 of 216	McBroom *et al*. (2006)
MK8A44	DH5α*nlpI*::Tn5, insertion after codon 118 of 294	McBroom *et al*. (2006)
MK7B29	DH5α*yieM*::Tn5, insertion after codon 427 of 483	McBroom *et al*. (2006)
MK5A31	DH5α*nlpA*::Tn5, insertion after codon 90 of 272	McBroom *et al*. (2006)
ADA600	MC4100 φλ(*rpoHP3::lacZ*)	Bianchi and Baneyx (1999)
MK496	ADA600/pWSK130	This work
MK480	ADA600 *degP*::Tn5, P1 donor MK11F26	This work
MK481	ADA600 *rseA*::Tn5, P1 donor MK5B7	This work
MK482	ADA600 *tolB*::Tn5, insertion after codon 188 of 430, P1 donor MK10E29	This work
MK483	ADA600 *tolA*::Tn5, insertion after codon 79, P1 donor MK5A43	This work
MK497	ADA600 *nlpI*::Tn5, P1 donor MK8A44	This work
MK524	ADA600 *nlpA*::Tn5, insertion after codon 90 of 272, P1 donor MK5A31	This work
MK527	ADA600 *ypjA*::Tn5, insertion after codon 165 of 1569, P1 donor MK4A31	This work
MK532	ADA600 *yieM*::Tn5, P1 donor MK7B29	This work
MK533	ADA600 *wzxE*::Tn5, insertion after codon 256 of 416, P1 donor MK1F40	This work
MK534	ADA600 *ompR*::Tn5, insertion after codon 47 of 239, P1 donor MK4E44	This work
MK535	ADA600 *ompR*::Tn5, insertion after codon 96 of 239, P1 donor MK10F34	This work
MK557	ADA600 *degS*::Tn5, P1 donor MK6D31	This work
MK559	ADA600 *degS*::Tn5, P1 donor MK6D31, wild-type σ^E^ activity	This work
MK600	ADA600 *ponB*::Tn5, insertion after codon 363 of 844, P1 donor MK7E17	This work
MK601	ADA600 *tatC*::Tn5, insertion after codon 76 of 258, P1 donor MK7C1	This work
MK602	ADA600 *lysS*::Tn5, insertion after codon 432 of 505, P1 donor MK6F18	This work
MK603	ADA600 *degP*::Tn5, insertion after codon 54 of 474, P1 donor MK7A41	This work
MK604	ADA600 *nlpI*::Tn5, insertion after codon 208 of 294, P1 donor MK5G18	This work
MK605	ADA600 *pnp*::Tn5, insertion after codon 341 of 734, P1 donor MK9D4	This work
MK606	ADA600 *pepP*::Tn5, insertion after codon 16 of 441, P1 donor MK11A9	This work
MK607	ADA600 *pal*::Tn5, insertion after codon 11 of 173, P1 donor MK8F18	This work
MK608	ADA600 *glnA*::Tn5, insertion after codon 467 of 469, P1 donor MK9G12	This work
MK598	ADA600 *tolB*::Tn5, insertion after codon 269 of 430, P1 donor MK6F12	This work
MK599	ADA600 *ompC*::Tn5, insertion after codon 183 of 367, P1 donor MK6A33	This work
MK537	ADA600 *ompC*::Tn5, insertion after codon 298 of 367, P1 donor MK12E45	This work
MK536	ADA600 *waaG*::Tn5, insertion after codon 296 of 374, P1 donor MK10G32	This work
MK525	ADA600 *waaG*::Tn5, insertion after codon 21 of 374, P1 donor MK10H29	This work
CAG43248	MC1061 [*φλrpoH* P3::*lacZ*Δ(*lacY*)::*cat*] pJM100 P_LlacO-1_ *degS*, Kan^R^ Spec^R^	Alba *et al*. (2001)
MC4100	F^–^*araD139*Δ(*argF-lac*)U169 *rpsL150 relA1 deoC1 rbsR fthD5301 fruA25*λ^–^	Casadaban (1976)
CAG16037	MC1061 φλ(*rpoHP3::lacZ*)	Mecsas *et al*. (1993)
WBS164	MC4100 φ(*lamB-lacZX90*) Hyb42-1[λ p1(209)]	Snyder and Silhavy (1995)
MK644	WBS164 *nlpI*::Tn5, P1 donor MK8A44	This work
MK649	WBS164 *nlpA*::Tn5, P1 donor MK5A31	This work
TR51	MC4100 *cpxR1*::*spc*	Raivio *et al*. (1999)
TR530	MC4100 λRS88 (*spy-lacZ*)	Raivio *et al*. (2000)
TR886	MC4100 λRS88 (*spy-lacZ*) *baeR1::kan*	Raffa and Raivio (2002)
K1527	K561 *pspF*::mTn*10-tet*	Jovanovic *et al*. (1996)
MVA4	MC1061 φ(*pspA-lacZ*)	Gift from G. Jovanovic
MK786	MVA4 *pspF*::mTn*10-tet*, P1 donor K1527	This work
ATCC 14028	Wild-type *Salmonella enterica* ssp. *enterica* serovar Typhimurium	ATCC
TF962	ATCC 14028 *degP*::cat	Testerman *et al*. (2002)
Plasmids		
pBA175	WT cytochrome-b_562_ in pBAD33, Cm^R^, arabinose-inducible	Walsh *et al*. (2003)
pBA182	Cytochrome-YYF fusion in pBAD33, Cm^R^, arabinose-inducible	Walsh *et al*. (2003)
pCS20	*degP* in pCS19, Amp^R^, IPTG-inducible	Spiess *et al*. (1999)
pCS19	pQE60-derived vector with *lacI*^q^, Amp^R^, IPTG-inducible	Spiess *et al*. (1999)
pJM100	pACYC177 derivative with *lacI*^q^, Kan^R^	McCarty and Walker (1994)
pJH68	*malE* in pHDB67, Amp^R^, arabinose-inducible	Szabady *et al*. (2005)
pHDB67	pBAD33-derived vector, Amp^R^, arabinose-inducible	Szabady *et al*. (2005)
pWSK130	Low-copy vector, Kan^R^	Wang and Kushner (1991)

### β-Galactosidase assays

Strains were grown overnight at 37°C, subcultured to an OD_600_ of ∼0.03, grown at 37°C to an OD_600_ of 0.3, and 0.5 ml assayed (Miller, 1992). Assays at vesicle harvest were performed with 0.05 ml culture. All assays were done in duplicate at least twice.

### Vesicle production assay

Unless otherwise indicated, broth cultures were inoculated at a 1:1000 dilution and grown overnight at 37°C. Cells were pelleted (10 000 *g*, 10 min, 4°C) and resulting supernatants filtered (low protein binding Durapore membrane, 0.45 μm polyvinylidene fluoride, Millipore). Filtrates were centrifuged (38 400 *g*, 1 h, 4°C), pellets resuspended in Dulbecco's phosphate buffered saline with added salt (0.2 M NaCl), and filter-sterilized through 0.45 μm Ultra-free spin filters (Millipore). Proteins were boiled in Laemelli buffer, separated by SDS-PAGE, and stained with either Coomassie Blue (Fisher) or SYPRO Ruby Red (Molecular Probes). *E. coli* OMPs F/C and A and *Salmonella* porins were quantified by densitometry (NIH Image software), and cfu ml^−1^ determined. Vesicle production was normalized to cfu ml^−1^ and compared with the indicated control strain to determine relative vesicle production; *n* ≥ 2, error bars indicate standard error.

### Preparative methods

Outer membranes were prepared as described (Kesty and Kuehn, 2004). Total culture samples were equalized to OD_600_ and concentrated by precipitation with 20% trichloroacetic acid.

### DegS depletion assay

Cells from a saturated culture of CAG43248 grown with 1 mM IPTG were pelleted, washed twice in LB, and inoculated into fresh media with or without 1 mM IPTG at a 1:50 dilution (OD_600_ 0.03). Vesicles harvested after 4 h growth at 37°C were normalized to culture cfu ml^−1^. Whole cell lysates were prepared by washing cells twice with LB, resuspending pellets in equal parts 1% SDS in PBS and 2× Laemelli buffer, and boiling for 10 min. Samples were separated by SDS-PAGE and immunoblotted.

### Vesicle integrity assay

Periplasm prepared as described (Kesty and Kuehn, 2004) or vesicles pelleted from cell-free supernatants were loaded to an Optiprep (Greiner) gradient and subjected to equilibrium density centrifugation (McBroom *et al*., 2006). Fractions were removed sequentially from the top of the gradient and separated by SDS-PAGE. Proteins were detected by staining with Coomassie blue and immunoblotting.

### Survival assays

Strains were grown to an OD_600_ of 0.4, then exposed to 10% ethanol or 2.5 μg ml^−1^ polymyxin B sulphate (Sigma) for 2 h. For maltose induction of LamB-LacZX90, strains were grown to an OD_600_ of 0.4, washed twice, resuspended in fresh media and induced with 0.02% maltose (EM Science). Cfu ml^−1^ values were determined immediately prior to chemical addition and after 2 h of exposure.

### Electron microscopy

Bacteria for scanning electron microscopy were washed in 0.1 M sodium cacodylate buffer, applied to a poly L-lysine-coated coverslip, fixed with 2.5% glutaraldehyde, dehydrated, sputter coated and viewed on a Philips XL 30 ESEM at 30 kV. For thin-section transmission electron microscopy, cells were fixed, washed in 0.1 M sodium cacodylate buffer, fixed in 1% OsO_4_, dehydrated and infiltrated in LR White resin.

## Supplementary material

The following supplementary material is available for this article online:

Fig S1 Establishment of an experimental time frame during which DegS depletion cultures are viable.

This material is available as part of the online article from http://www.blackwell-synergy.com
