# Supplementary material for: Release of outer membrane vesicles by Gram-negative bacteria is a novel envelope stress response
Source: Mol Microbiol. 2007 Jan 1;63(2):545–58. doi: 10.1111/j.1365-2958.2006.05522.x (PMC1868505; doi:10.1111/j.1365-2958.2006.05522.x)
Supplement: Fig S1 — Establishment of an experimental time frame during which DegS depletion cultures are viable. [file mmi0063-0545-s1.pdf]

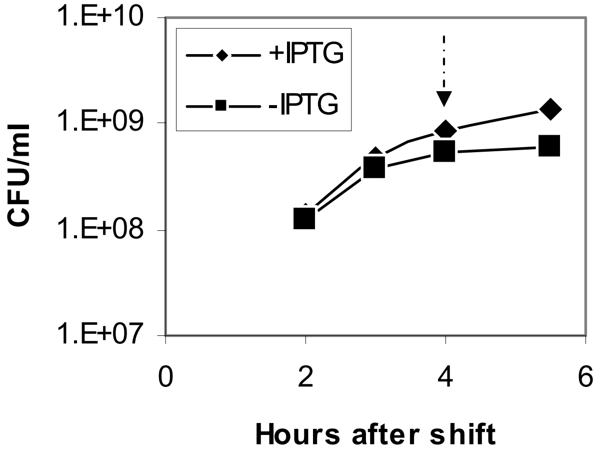

Figure S1. Establishment of an experimental time frame during which DegS depletion cultures are viable. Representative data showing growth characteristics of the DegS-depletable strain CAG43248 grown in the absence (-IPTG, DegS depleted) or presence (+IPTG, DegS expressed) of IPTG. Cells from a saturated culture grown in the presence of 1mM IPTG were washed twice in LB to remove IPTG, inoculated at a 1:50 dilution into fresh LB media with or without IPTG, and grown with shaking at 37°C. Viability was assessed by dilution plating and CFU/ml calculated. The arrow indicates the 4-hour time point at which relative vesicle production assays were conducted.
